# Daily functional electrical stimulation during everyday walking activities improves performance and satisfaction in children with unilateral spastic cerebral palsy: a randomized controlled trial

**DOI:** 10.1186/s40945-015-0005-x

**Published:** 2015-07-18

**Authors:** Dayna Pool, Jane Valentine, A. Marie Blackmore, Jennifer Colegate, Natasha Bear, Katherine Stannage, Catherine Elliott

**Affiliations:** 1 Department of Physiotherapy and Paediatric Rehabilitation, Princess Margaret Hospital for Children, Roberts Road, Subiaco, 6008 Perth, Australia; 2 Department of Paediatric Rehabilitation, Princess Margaret Hospital for Children, Perth, Australia; 3 The Centre for Cerebral Palsy, Perth, Australia; 4 Department of Occupational Therapy and Paediatric Rehabilitation, Princess Margaret Hospital for Children, Perth, Australia; 5 Department of Orthopaedics, Princess Margaret Hospital for Children, Perth, Australia; 6 Faculty of Health Science, Curtin University of Technology, Perth, Australia

**Keywords:** Cerebral palsy, Unilateral spastic cerebral palsy, Spastic hemiplegia, Randomized controlled trial, Canadian occupational performance measure, Functional electrical stimulation, Activity, Participation, Satisfaction, Gait

## Abstract

**Background:**

The aim of this paper is to determine whether daily functional electrical stimulation (FES) is effective in improving self-perceptions of individually identified mobility performance problems in children with unilateral spastic cerebral palsy (USCP). We hypothesized that children receiving 8 weeks of FES treatment would have higher scores for self-perceived performance and satisfaction on the Canadian Occupational Performance Measure (COPM) for individually identified priorities than children not receiving FES.

**Methods:**

Thirty-two children (mean age 10 y 8 mo SD 3y 3mo) with USCP and a Gross Motor Function Classification System I or II were randomly assigned to the FES treatment group (8 weeks of daily FES) and control group (usual treatments). Participants were assessed at baseline (week 0), post treatment (week 8) and 6 weeks follow-up (week 14). The primary outcome measures were self-perceived scores for performance and satisfaction of child- and parent-identified priorities assessed using the COPM post treatment and at follow-up. The secondary outcome measures were the categorization of the performance problems from the COPM and self-report responses according to the International Classification of Functioning Child and Youth version (ICF-CY). This was clinically important because an understanding of mobility performance problems for children with USCP is needed for family-centred service planning.

**Results:**

Performance scores (mean difference 1.6, 95 % CI 0.1 to 3.2, *p* = 0.034) and satisfaction scores post treatment (mean difference 2.4, 95 % CI 0.5 to 4.2, *p* = 0.004) were significantly higher in the treatment group than in the control group. There were no significant differences between the groups for performance scores at follow up, however there was a significant difference between the groups for satisfaction (mean difference 1.9, 95 % CI 0.1 to 3.8, *p* = 0.03) in favour of the treatment group. Priorities were identified across all levels of the ICF-CY but were most commonly identified in the activity and participation domains of the ICF-CY (79.5 %).

**Conclusions:**

Daily FES applied during everyday walking is effective in addressing self-perceptions of individually identified priorities by improving the performance and satisfaction of functional skills after treatment.

**Trial registration:**

Australian New Zealand Clinical Trials Register ACTRN12614000949684. Registered 4 September 2014.

## Background

Cerebral palsy describes a group of permanent motor dysfunctions caused by non-progressive damage to the developing brain. It is often accompanied by secondary musculoskeletal impairments that can exacerbate activity limitations [18]. Unilateral spastic cerebral palsy (USCP) is the most common topographical presentation of cerebral palsy [1]. Most children with USCP are classified as having a Gross Motor Function Classification System (GMFCS) level of I or II [13]. This means that though children are independently ambulant, they still have limitations when walking in the community. This is largely attributed to the common lower limb secondary musculoskeletal impairments that are exacerbated with growth in children with USCP. These include a combination of gastrocnemius muscle spasticity, contracture, ankle dorsiflexion weakness and poor ankle selective motor control. The combination of these impairments limits effective foot clearance during the swing phase of gait and can cause tripping or falling when walking [24].

Functional electrical stimulation (FES) applied to the ankle dorsiflexors during the swing phase of gait can be used to address problems with foot clearance. FES refers to the application of neuromuscular electrical stimulation to muscles that may not be able to contract voluntarily within a task-specific functional activity such as walking [12]. An electrical current is used to produce an involuntary muscle contraction by inducing an action potential through the placement of two electrodes over the surface of the skin above the target skeletal muscle [17].

Evidence to support the effectiveness of FES in children with cerebral palsy has been increasing over recent years, particularly because devices suitable for children can now be purchased commercially. They are also more user-friendly, enabling FES to be managed by children and families in the community [11, 14, 15]. Common outcome measurements used to evaluate the effectiveness of FES have been focused mainly on the body structure and function level, which include range of motion, spasticity, strength, muscle volume and gait mechanics [7, 15, 19]. Although these measures have provided useful clinical information, they are not able to indicate how FES impacts the performance of individually specific daily activities in the community and the satisfaction of the user. Given that the application of FES during walking enables the intervention to be applied in the community, there is also a need to determine its effectiveness within these environments.

The Canadian Occupational Performance Measure (COPM) is a valid and reliable client-centred instrument that provides the opportunity to evaluate self-perceived effectiveness of treatment whilst considering the individually specific environment in which it is performed in [2, 5, 22]. Hence the COPM will also be used to evaluate the changes in the self-perception of performance and satisfaction of individually identified priorities following daily FES during everyday walking activities.

The aim of this paper is to determine whether FES in children with USCP is effective in improving self-perceptions of individually identified mobility performance problems when compared to children receiving usual treatments. We hypothesized that children receiving 8 weeks of FES treatment would have higher scores for self-perceived performance and satisfaction on the COPM for individually identified priorities than children not receiving FES. We also hypothesized that children who received FES treatment would continue to have higher scores for self-perceived performance and satisfaction at follow-up than children not receiving FES. The secondary aim of this study was to explore the mobility performance of children with USCP by employing the International Classification of Functioning Child and Youth version (ICF-CY) framework. This is clinically important because an understanding of mobility performance problems for children with USCP is needed for family-centred service planning.

## Methods

### Study design

The study design was a randomized controlled clinical trial of daily FES during every day walking activities to the ankle dorsiflexors compared with usual treatments (control group).

### Participants

Participant inclusion criteria (Table 1) included: USCP, Gross Motor Function Classification System [13] level and Winters Gage and Hicks Classification [24] of I or II, age 5 to 18 years, at least 5 degrees passive ankle dorsiflexion and full knee extension, ability to co-operate with the assessment procedures, and willingness to use FES daily over 8 weeks. The schedule for study commencement was dictated by current clinical care involving botulinum toxin type A injections that is routinely delivered every 6 months. With the exception of 4 children who do not have routine botulinum toxin type A injections (2 children in the treatment group and 2 children in the control group), all remaining children have 6 monthly botulinum toxin type A injections. For these children, baseline measures commenced 3 months after injections which is widely accepted to be after the peak technical response due to motor end plate regeneration [8]. Participants were excluded if they had orthopaedic surgery performed on the affected side less than 12 months before the study commenced, if they had orthopaedic metalware at the site of stimulation, or if they had an uncontrolled seizure disorder.

**Table 1:** Inclusion and exclusion criteria

Inclusion Criteria	Exclusion Criteria
• Passive dorsiflexion range of affected ankle of at least 5°	• History of uncontrolled seizure disorder
• Full passive knee extension bilaterally	• Orthopaedic lower limb surgery on the affected side in the past 12 months
• Dynamic popliteal angle of no more than 45°	• Orthopaedic metal ware at the site of electrical stimulation
• Able to cooperate with assessment procedures	• Botulinum toxin in lower limb in the past 3 months
• Willing to use the Walk Aide ® at least 4 h a day, 6 days a week for 8 weeks	
• GMFCS I or II, unilateral spastic cerebral palsy (with or without dystonia)	
• Winters Gage and Hicks gait classification of Type I or II	
• Aged between 5 and 18 years	

Participants were referred from Physiotherapists and Paediatric Rehabilitation consultants between June and July 2013 from clinics of the Cerebral Palsy Mobility Service at Princess Margaret Hospital for Children and The Centre for Cerebral Palsy in Perth, Australia. The trial commenced in August 2013 with the final assessments completed by April 2014. Ethics committees at Princess Margaret Hospital for Children and The University of Western Australia approved the trial. The committees’ recommendations were adhered to. Written and informed consent for participation and publication was obtained from all participants. This trial was retrospectively registered (ACTRN12614000949684). However no changes were made to the protocol that was approved by the ethics committees.

### Procedure

An initial appointment was firstly arranged by the first author (DP): a Physiotherapist, to determine FES tolerance and discuss the study protocol. Randomization to either the FES or control group was achieved through a coin toss, by an individual uninvolved with the study once 2 matched participants were enrolled. Matched participants were of the same GMFCS level, and were within 2 years of age for children aged between 5 and 10, and within 6 years for children aged between 11 and 18. This method was applied to improve the homogeneity of each group in terms of age and gross motor function.

### Outcome measures

The primary outcome measures were self-perceptions of performance and satisfaction of individually prioritised mobility performance problems derived from the COPM. The secondary outcomes were the categorization of the priorities identified in the COPM and self-reported parent/participant observations post treatment (FES group only at post treatment) into the domains of the ICF-CY [25].

### Canadian occupational performance measure

A single interviewer (JC): an Occupational Therapist performed the COPM at all time points and was blinded to group allocation. At baseline, the interviewer assisted the child and family to identify occupational performance problems in the areas of self-care, productivity or leisure. Once they had identified these problems, they were written positively as goals, which participants and their parents then prioritised by importance on a scale from 1 to 10 (10 indicating greater importance). Scores out of 10 for self-perceived performance and satisfaction were then obtained from each participant (if the child was 12 years or older) or parent (if the child was under 12 years). The scores were summed and averaged over the number of priorities identified to produce two overall scores out of 10 for each participant: one for performance and one for satisfaction. At post-treatment and follow-up, participants were blinded to their previous ratings in order to limit potential bias [23]. A 2 point change in score on the COPM is considered to be clinically meaningful [23].

### Participant and parent self-report

At post-treatment, participants and their parents were asked for written comments in answer to the question: “Have you noticed any changes in yourself since using the Walk Aide?” Again, parents answered on behalf of children under 12 years.

### FES intervention

Participants in the FES group received the FES device after the baseline assessment. The Walk Aide® (Innovative Neurotronics, Austin, TX, USA) is a small (8.2 cm x 6.1 cm x 2.1 cm, 87.9 g) device that delivers asymmetrical biphasic surface electrical stimulation (ES) in a synchronized manner to stimulate active dorsiflexion of the ankle during the swing phase of gait. The Walk Aide® is attached to the participant’s leg by a cuff and sits just below the knee on the affected side. During a gait cycle, the Walk Aide® stimulates the common peroneal nerve, which innervates tibialis anterior and other ankle dorsiflexors (extensor digitorum longus, peroneus tertius and extensor hallucis longus). Ankle dorsiflexion was achieved by the placement of one electrode over the fibular head to stimulate the peroneal nerve and the other electrode on the motor end point of tibialis anterior. Pulse width was set to a maximum of 300 microseconds (μs) and frequency was set at 33 hertz (Hz). Users could adjust intensity (mA) using a dial on the device. The Walk Aide’s® tilt sensor was individually synchronized and saved on the device so that the stimulation to the ankle dorsiflexors could occur immediately after toe-off, remaining activated during the swing phase of gait until initial contact.

Weekly to fortnightly community physiotherapy home and school visits were provided for parents and teachers/education assistants to support FES use in different environments whilst ensuring correct use of the cuff and accurate electrode placement. Participants were asked to change electrodes every two weeks. Any adverse events were to be reported immediately (via text message or email) to the first author (DP) in order to ensure follow-up in a reasonable time frame. Participants were asked to use the FES device for at least 4 h a day, 6 days a week during the 8-week treatment period. This was monitored through the usage log on the device itself. To enable participants an opportunity to accommodate to the device, they were asked to build up gradually to the required dosage over the first week. The 8-week treatment period and 6-week follow-up period was chosen based on the results from our pilot study [14] as well as around current clinical care on the use of botulinum toxin injections (essentially so that the study duration would not be interrupted by botulinum toxin injections in an effort to minimize the confounding effect of the injections to overall outcome).

Participants in the treatment group did not wear their ankle foot orthosis (AFO) either during the FES treatment phase or in the follow-up phase but where appropriate, were provided with customized in-shoe orthosis at the commencement of the study to support foot posture and accommodate for leg length discrepancies. Participants in the control group were asked to continue with their usual orthotic protocol. To maintain consistent contact with the participants, fortnightly home or school visits were also provided for each participant in the control group.

### Statistical analysis

Normality was established for the COPM scores through examining distributional plots, Q-plots and the Shapiro-Wilk Test. Means and standard deviations were reported for each group for each phase. Within group differences were assessed for a clinically meaningful change i.e. 2 point score change [23] from baseline. Between group differences were examined using a repeated measures ANOVA to account for the correlation between repeated measures over time. Post-hoc Tukey’s test was applied if a main effect for group and time or an interaction of these was found, enabling adjustments for multiple comparisons and calculation of mean differences and 95 % confidence intervals. Assumptions for the repeated ANOVA were examined and met.

Statistical significant was accepted as *p* < 0.05. All statistical analyses were performed using STATA version 12.1 (TestCorp, Texas).

To further explore the performance components of the identified priorities, each priority was analysed using 2 methods. Firstly, each priority was categorized by using the occupational performance model i.e. relating to occupational performance roles, areas or components [9, 10] which secondly, facilitated the translation to the ICF-CY to identify which domain or domains it addressed [16]. Two of the authors (DP and AMB) completed this process with 92 % agreement (differences resolved by discussion).

Self-reported responses concerning overall impressions of the FES device were compiled, thematically analysed and categorized using the ICF-CY by 2 of the authors (DP and AMB). Examples are presented verbatim.

## Results

**Fig. 1:**
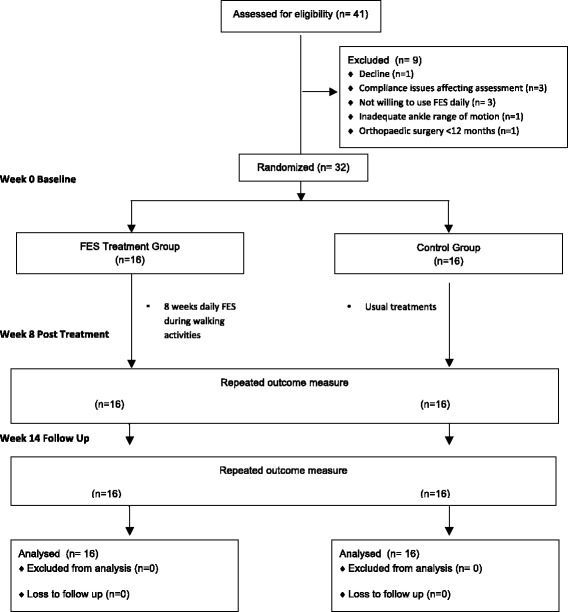
Flow of participants through the trial. FES, Functional Electrical Stimulation

Thirty-two children, mean age 10 y 8 mo (range 5 y 5 mo – 18 y 1 mo) with USCP GMFCS level I or II were recruited for the study. All participants had a Winters Gage and Hicks gait classification of I or II indicating foot clearance problems during walking gait. All participants completed the study in their original group allocation. There were no missing data (Fig. 1).

There were no clinically meaningful differences between the groups at baseline on the COPM (Table 2). Tests for normality showed that COPM scores were approximately normally distributed for performance (Shapiro-Wilk Test *p* = 0.133, Skewness −0.79, Q-plot normal) and satisfaction (Shapiro-Wilk Test *p* = 0.49, Skewness −0.36, Q-plot normal). Participants used the FES daily for a mean of 6.2 (SD 3.2) h over the 8-week intervention period. All participants had a frequency set at 33Hz and pulse width ranging from 25-100 μs. There were no reported unintended effects or adverse events using the FES device.

**Table 2:** Baseline characteristics of participants

	Treatment	Control	*p* value
Weight (kg)	38.5 (15.2)	37.4 (15.9)	0.850^a^
Gender	Male: 9	Male: 8	
	Female: 7	Female: 8	
Side of hemiplegia	Right: 11	Right: 12	0.950^a^
	Left: 5	Left: 4	
GMFCS	I: 10	I: 10	
	II: 6	II: 6	
WGH	I: 1	I: 0	
	II: 15	II: 16	
Age	10y 11mo	10y 5mo	
	(3y 10mo)	(2y 8mo)	
COPM			
Performance	3.97 (1.42)	4.25 (1.42)	0.978^a^
Satisfaction	4.36 (1.69)	4.18 (0.99)	0.719^a^

^a^ independent samples t test; GMFCS, Gross Motor Function Classification System, *WGH* Winters Gage and Hicks, *COPM* Canadian Occupational performance Measure

### Primary outcome: COPM

There was a significant main effect for group (performance *p* < 0.001; satisfaction *p* < 0.001), time (performance *p* < 0.001; satisfaction *p* < 0.001) and for interaction of group and time (performance *p* = 0.003; satisfaction *p* = 0.002). Post treatment, performance scores (mean difference 1.6, 95 % CI 0.1 to 3.2, *p* =0.034) and satisfaction scores (mean difference 2.4, 95 % CI 0.5 to 4.2, *p* = 0.004) were significantly higher in the treatment group than in the control group. At follow-up, there were no significant differences between the groups for performance scores (mean difference 1.2, 95 % CI −0.4 to 2.8, *p* = 0.224). However, there was a significant difference between the groups for satisfaction (mean difference 1.9, 95 % CI 0.1 to 3.8, *p* = 0.030), again in favour of the treatment group.

**Fig. 2:**
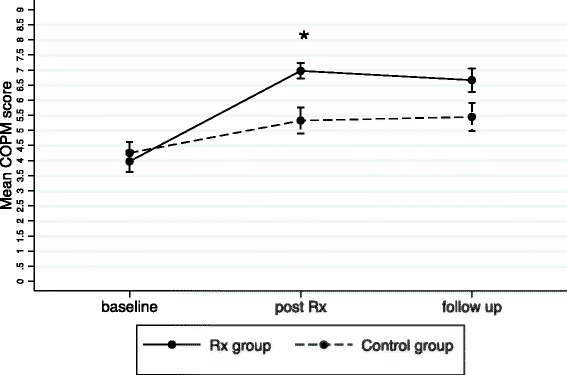
Mean and standard error of COPM Performance at baseline, post 8 weeks Rx (treatment) and 6 weeks follow‐up; *significant difference between groups *p*<0.05

**Fig. 3:**
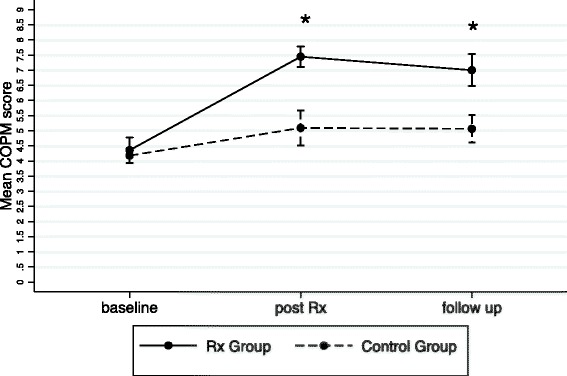
Mean and standard error of COPM satisfaction at baseline, post 8 weeks Rx (treatment) and 6 weeks follow‐up; *significant difference between groups *p*<0.05

From the baseline performance score in the treatment group (3.97, SD 1.42), there were clinically meaningful changes (i.e. >2 point change) post treatment (6.97, SD 1.04) and at follow-up (6.66, SD 1.57). From the baseline satisfaction score in the treatment group (4.36, SD 1.69), there were also clinically meaningful changes post treatment (7.45, SD 1.34) and at follow-up (6.99, SD 2.11). There was a trend for the control group having higher scores at post treatment and follow-up than at baseline for performance and satisfaction but these changes were not clinically meaningful. These results are shown graphically in Figs. 2 and 3.

### Secondary outcome: ICF-CY classification of priorities

Participants in the study identified 1 to 3 priorities each. There were a total of 80 individual priorities for the 32 participants. Some of the priorities involved more than 1 domain on the ICF-CY. For example, one of the priorities was “to walk consistently with a heel to toe pattern to improve my symmetry (so I don’t have to wear an AFO)”. This included 2 parts involving (a) improving walking mechanics (activity) (b) not need an AFO (personal). Hence, when the original 80 priorities were divided into their component parts, there were 122 specific priorities. These 122 priorities were categorized into body structure and function, activity and participation domains as well as personal and environmental factors. As shown in Table 3, 17 % of the priorities were directed towards the need to improve impairments in body structure and function, 49 % were directed towards improving functional mobility in the activities domain and 31 % were directed towards improving community mobility and active recreation in the participation domain.

**Table 3:** Breakdown of the number (out of a total of 122) and percentage of ICF-CY domains (alongside the occupational performance components, areas and roles) in the identified priorities

Body Structure and Function Performance Components	N	%
Biomechanical		
Strength	15	
Balance	4	
ROM	1	
Sensory	1	
Leg pain	1	
	Sub Total	17 %
Activities Occupational Performance area		
Functional Mobility		
Improve walking mechanics	32	
Improve walking endurance	6	
Improve running	14	
Improve high level gross motor skills	7	
	Sub Total	49 %
Participation		
Occupational performance; role competence		
Community mobility		
Reduce falls	7	
Reduce trips	4	
Active Recreation		
Keep up with friends	10	
Improve sport performance	17	
	Sub Total	31 %
Environmental/Personal Factors		
Wear certain kinds of shoes	3	
Not need AFO	1	
	Sub Total	3 %

### Secondary outcome: ICF-CY classification of self-reported changes in treatment group

ICF-CY analysis identified 5 major themes: (a) improved running and walking (activity, n = 13); (b) improved comfort with more options to wear different shoes (personal factor, n = 6) with comments such as “the walk aide means less blisters on my feet, easier to put on shoes*”* and *“*her dad got her ‘girlie’ shoes and they stay on her feet – really pleased*”;* (c) reduction in trips and falls (participation, n = 4), (d) improved confidence (personal factor, n = 4) and; (e) increased foot awareness (body structure and function, n = 2) with comments such as “feel more aware of your foot placement when wearing the walk aide*”* and “I can feel when it raises my toes when walking”.

The comments also yielded some disadvantages of the Walk Aide® (n = 2) which included problems with the size and difficulties in getting clothing to over it, causing the cuff to “fall apart quite often”. Three of the participants did not wish to continue wearing Walk Aide® beyond the study period because of difficulties with accurate placement, bulkiness, problems with wearing school uniforms (stockings or leggings), and difficulties in attaining a good fit owing to the cuff sliding down the leg during walking. The remaining 13 participants in the FES group continued to use the Walk Aide® either as an AFO replacement or as an adjunct to their AFO protocol.

Participant 10 wrote a more detailed account of her experiences post treatment:

“Although compared to many other cases, my CP is quite mild, it has had quite an effect on me over the years; mentally, physically and emotionally…. To be honest I wasn't very keen on it in the beginning; I felt like it added to the things that made me different… but as the study progressed it quickly became the thing that drew me closer to my peers. Emotionally, I struggled with feeling different or out of place; having to wear splints or orthotics, but through the use of the Walk Aide, I began to feel more confident and enthusiastic to do the things I had to do to maintain the physical effects of the Walk Aide. The Walk Aide for me, reduced, in fact eliminated my pain (foot, leg and low back), boosted my confidence, gave me the ability to wear shoes like thongs in summer (one of my goals), increased my energy levels, and gave me the ability to walk long periods of time without growing weak or sore.”

## Discussion

The FES group achieved significantly higher scores for self-perceived performance and satisfaction on the COPM at post treatment than the control group. This supports the first hypothesis, that FES is effective in improving self-perceived performance and satisfaction of individually identified mobility performance problems in children with USCP. There has been limited support for the efficacy of FES, particularly in regard to the activity and participation domains of the ICF-CY. This has in part, been attributed to the limited inclusion of valid and reliable activity and participation outcome measures [3, 4]. Therefore, these results not only provide unique evidence supporting the effectiveness of FES on activity and participation but also, that the results are consistent with current knowledge on the effectiveness of FES on the main lower limb impairments observed in children with USCP. Currently, the literature supports that FES can improve selective motor control, range of movement, spasticity, strength and ankle kinematics during gait [11, 14, 15]. Therefore, it appears that by implementing FES during daily walking activities, the main impairments affecting gait in children with USCP are addressed alongside quantifiable functional benefits reflected in the activity and participation domains.

Children who received FES treatment continued to have higher scores for self-perceived satisfaction, but not for performance when compared to children not receiving FES at follow-up. This partially supports the second hypothesis. This is consistent with current findings documenting that the effects of FES on muscular adaptations are use-dependent [7]. This provides a plausible explanation for why the self-perceived performance scores were no longer significantly improved in the treatment group when compared to the control group at the 6 week follow-up. However, this result also suggests that a period of no FES (to a maximum of 6 weeks) can be incorporated into the management plan for children with USCP without significant detriment to the satisfaction of users. This may be advantageous because of the potential to develop dependence on the external stimulus replacing the internal control of movement [6]. Given that self-perceived performance scores were no longer significantly higher in the treatment group than in the control group at follow-up, a non-use period greater than 6 weeks would not be recommended. Alternating between FES use and non-use, as adopted in this study, could be implemented to suit individual and family needs such as planning around holidays, school camps and seasons. Further study is warranted to determine whether extending this regimen would maintain the effects reported in the present study.

The secondary analysis of the priorities identified in the COPM demonstrated that the majority of occupational performance problems for children and parents were related to functional mobility activities, community mobility and active recreation participation. However in some instances, children and parents also identified specific impairments in body structure and function. Although the construct of the COPM facilitates the identification of priorities more relating to activities and participation, we included this data to reflect the priorities of children and their parents. The inclusion of priorities in this domain highlights that children and their parents understand the body structure and function components that influence performance and for Physiotherapists, the value of addressing them in treatments. However, it also reinforces the importance of clear and sensitive communication from clinicians, realizing the influence of language on the priorities of children and their parents.

In some instances, multiple domains were included within each priority, reflecting the complexity and breadth of outcome measures that would be required in order to capture and quantify potential effects of treatment. Previous FES studies have reported discrepancies between the outcomes of objective clinical outcome measures and the more favourable parent reports [14, 20]. The complexity of priorities may explain the discrepancy between clinical objective measures and parent report, because the objective outcome measures emphasized body structure and function assessed within a clinical environment. Hence the outcome measures did not align closely with the priorities of children and parents, which often emphasized activities and participation in their own environment and community.

The self-report also provided some additional insights to (previously unreported) effects of FES after 8-weeks of use. These included reports of improved confidence, improved foot awareness, and improved range of footwear. The results of this study support the effectiveness of community applied FES to improve self-perceived individually identified priorities, particularly when they involve the performance of functional mobility, community mobility and active recreation in children with USCP.

### Considerations and recommendations for the use of daily FES

Participants and their parents described some disadvantages to wearing the FES device. Their comments indicated that acceptance of the FES device goes beyond mere biomechanical physical and compliance requirements. Because the FES device needed to be strapped directly over skin, there were issues with clothing, in particular leggings or stockings for school uniforms. For younger children, the combination of the cuff and device were bulky, and parents struggled to find clothing to fit over it. Also for younger children, the cuff fitting was an issue, as it would slide down the leg during walking and running. Older children usually managed these problems, but younger children needed to have adequate support at home and school. When prescribing the Walk Aide®, it is important to consider the individually specific environmental factors that may affect treatment. Therefore, providing information and education to people involved in the child’s care is essential. This highlights the importance of ensuring that community therapy services are available and in place prior to considering this intervention. However it should be noted that the Walk Aide® is still essentially a device that was specifically designed for adults. Though small cuffs have been recently available, the size of the Walk Aide® continues to be a potentially limiting factor for patient selection. Further investment into the technology and fit of this device is recommended.

Future studies should consider the cost effectiveness of this intervention. Although the cost-effectiveness of FES in the adult population has been supported [21], this has not been evaluated in children with USCP. For some children, it may be appropriate for FES to replace the use of AFOs. However, in other children, FES may be an adjunct to current therapy and AFO intervention. Evidently, this influences costs and should be evaluated further. Further work is also warranted to develop a questionnaire that would be appropriate for younger children to provide further perspectives on the effectiveness of FES treatments.

There are some limitations to note. Although qualitative self-report provided insights into the FES experience, it was unstandardized, and so the comments must be considered with some caution. Also, the outcomes of the intervention were dependent on family and school support. This could not be controlled and may have varied across the participants.

## Conclusions

Daily FES during everyday walking activities improves self-perceptions of individually identified priorities, particularly involving the performance of activities, community mobility and active recreation in children with USCP. Alternating between a period of use and non-use may be appropriate without detriment to the satisfaction of the user, and this may be beneficial to suit family needs. The role of community therapy is also highlighted for the education and training of both families and teachers so that this intervention can be successfully implemented within each child’s own relevant environments.

## Abbreviations

FES: Functional Electrical stimulation
USCP: unilateral spastic cerebral palsy
COPM: Canadian Occupational Performance Measure
ICF-CY: International Classification of Functioning Child and Youth version
AFO: ankle foot orthosis
SD: Standard Deviation
CI: Confidence Interval
IQR: interquartile range
Rx: treatment
CP: cerebral palsy
GMFCS: Gross Motor Function Classification System
